# Supplementary material for: Lower-skilled occupations face greater upskilling pressure in U.S. job ads
Source: Nat Commun. 2025 Dec 31;17:1237. doi: 10.1038/s41467-025-67992-y (PMC12864811; doi:10.1038/s41467-025-67992-y)
Supplement: Supplementary file 1 — Supplementary Information [file 41467_2025_67992_MOESM1_ESM.pdf]

Supplementary Information for

Lower-skilled Occupations Face Greater Upskilling Pressure in U.S. Job Ads

Di Tong, Lingfei Wu, James A. Evans

Correspondence to: jevans@uchicago.edu (J.A.E.)

CONTENTS

1. Supplementary Note 1: Representativeness of Job Ads Data Collected by Lightcast
2. Supplementary Note 2
    - 2.1 Formula and Replication of Deming and Noray's (2020) Job Skill Change Measurement
    - 2.2 Occupational Skill Change Measurements in Figure 1c and d Illustration
    - 2.3 Cluster Approach to Measure Occupational Skill Change
    - 2.4 Embedding Validation: Internal Validation
    - 2.5 Embedding Validation: Pre-trained LLM
    - 2.6 Embedding Validation: Factor Analysis
    - 2.7 Identifying the Direction of Occupational Skill Change with Skill Atoms
3. Supplementary Note 3
    - 3.1 Job Zone and Skill Change
    - 3.2 Occupational Skill Change as the Most Significant Skill Transition
    - 3.3 Upskilling of Lower-Skilled Jobs
    - 3.4 Meaning and Significance of Occupational Skill Change: Re-Educational Costs
    - 3.5 Labor Market Tightness and Skill Change
    - 3.6 Robustness Check: Controlling for Employer Concentration, within-occupation job role homogeneity, and its changes
    - 3.7 Robustness Check: Different Job Content Scope with Skill Weights
4. Supplementary Figures
    - 4.1 Figure S1.Validation of Lightcast data representativeness
    - 4.2 Figure S2. Cluster approach to measure occupational skill change
    - 4.3 Figure S3. T-SNE visualization of skill vectors
    - 4.4 Figure S4. T-SNE visualization of occupation vectors
    - 4.5 Figure S5. Occupation skill vectors and title word vectors comparisons
    - 4.6 Figure S6. Occupations' re-skilling direction illustrated by skill atoms
    - 4.7 Figure S7. Job zone and skill change
    - 4.8 Figure S8. Upskilling of low-skilled jobs towards high-skilled jobs
    - 4.9 Figure S9. Distribution of number of added skills by occupation
    - 4.10 Figure S10. Education Cost of Marginal Skill Vector Distance between Jobs
    - 4.11 Figure S11. Unemployment rate and job skill change
    - 4.12 Figure S12. Mapping U.S. Labor Markets' Upskilling Pressure Through Automation Risk
5. Supplementary Tables
    - 5.1 Table S1. Explaining job skill change variation with skill complexity, measuring change with the most significant skill transition
    - 5.2 Table S2. Example of job posts pairs used for predicting re-education cost
    - 5.3 Table S3. Predicting education year difference with skill distance
    - 5.4 Table S4. Explaining job skill change variation with skill complexity
    - 5.5 Table S5. Explaining job skill change variation with employer and market size, within firm-region-occupation change
    - 5.6 Table S6. Explaining job skill change variation with employer and market size, firm-region

overall change
5.7 Table S7. Explaining job skill change variation with skill complexity, different job content scope and skill weights
5.8 Table S8. Explaining job skill change variation with employer and market size (within firm-region-occupation change), different job content scope and skill weights
5.9 Table S9. Explaining job skill change variation with employer and market size (firm-region overall change), different job content scope and skill weights

1. Supplementary Note 1: Representativeness of Job Ads Data Collected by Lightcast

Lightcast data could be biased for multiple reasons. They may include duplicate job ads and oversample higher skill jobs which are more likely than lower skill jobs to appear in online posts. Moreover, among the analyzed job ads, only 50% specified educational requirements and 17% listed salary. To address these concerns, we have compared Lightcast data against the 2010 and 2018 Occupational Employment Statistics (OES) assembled by U.S. Bureau of Labor Statistics (BLS) in job demand / employment, education requirement, and salary for the entire sample of occupations. We confirm that these two data sources are highly consistent in all three variables. Specifically, we find the Pearson correlation coefficient r~0.8 (p<0.001) for labor market share (Fig. S1a), r~0.8 (p<0.001) for salary (Fig. S1b), and r~0.9 (p<0.001) for education (Fig. S1c).

In Fig. S1a, each dot represents a 6-digit SOC occupation. 786 Occupations can be matched between Lightcast job post data and BLS-OES data in 2018; 777 occupations can be matched between the two datasets in 2010. The X axis denotes the log value of Lightcast vacancy post number for each occupation. The Y axis denotes the log value of BLS estimated employment for each occupation. The Pearson correlation between the log of Lightcast occupational vacancy post number and the log of BLS occupational employment is 0.8 (p<0.001) in 2018 and 0.76 (p<0.001) in 2010. The magnitude difference between BLS occupational employment data and Lightcast occupational demand data could be attributed to the fact that employment includes incumbent workers not reflected in labor demands, and that not all jobs hire workers through online job ads and a single job post may seek to hire more than one worker.

Fig. S1b presents 772 occupations that can be matched between Lightcast and BLS-OES datasets in 2018; and 761 occupations that can be matched between the two datasets in 2010. X and Y axes denote the log value of Lightcast and BLS average annual median salary for each occupation, respectively. The Pearson correlation between the log of Lightcast and BLS occupational annual median pay is 0.87 (p<0.001) in 2018 and 0.83 (p<0.001) in 2010.

In Fig. S1c, each dot represents an occupation in 2018, summing to 682 matches between Lightcast entry education requirement data and BLS education and training assignments by detailed occupation data in 2018. The X axis denotes the average Lightcast entry education year requirement for each occupation in 2018. The Y axis denotes the BLS estimated typical entry education year for each occupation in 2018. Here 12 refers to High school diploma or equivalent; 14 to Associate's degree; 16 to Bachelor's degree; 18 to Master’s degree; 21 to Doctoral or professional degree. Note that in Lightcast’s job posts, education year could only take a value from the set {12, 14, 16, 18, 21}, denoting different degrees {“high school”, “associates”,

“bachelors”, “masters”, “doctorate”}. Nevertheless, because we calculated the average education year from all job posts for each occupation, the occupational average education year could take on value between those in the set. BLS, on the other hand, only provides entry degree level for each occupation: “No formal educational credential”; “High school diploma or equivalent”; “Some college”, “No degree”; “Postsecondary nondegree award”; “Associate's degree”; “Bachelor's degree”; “Master's degree”; “Doctoral or professional degree”. In order to compare the two datasets, occupations with “No formal educational credential” are left out; “Some college”, “No degree” as well as “Postsecondary nondegree award” are coded as 12 (the same as “High school diploma or equivalent”). The Pearson correlation between Lightcast and BLS 2018 occupational entry education level is 0.92 (p<0.001).

We also verify the Two-step Deduplication Process that Lightcast used[1], in which the key component is to build advanced parsing engine to extract and normalize a number of data elements from each job listing, including job title, job ID, source, posting date, employer name, location, job description text, etc., and then use these variables to screen for duplicates. In our data analysis, we did not find job posts that duplicated all of these fields.

2. Supplementary Note 2

2.1 Formula and Replication of Deming and Noray’s (2020) Job Skill Change Measurement

As is demonstrated in equation 1, Deming and Noray (2020)[2] measure the skill content change of occupation o as the sum of the absolute value of difference in share for each skill from 2007 to 2019, in which a given skill s’s share of occupation o in year t is defined as the proportion of o’s job ads that require s in year t. In equation (1)a, $\left(\frac{\text{skill}_o^s}{\text{JobAds}_o}\right)_{t1}$ is the number of job ads of occupation o in year $t1$ that require skill s divided by the number of job ads of occupation o in that year. Similarly, $\left(\frac{\text{skill}_o^s}{\text{JobAds}_o}\right)_{t0}$ is this share of skill s for occupation o at $t0$. The absolute value of the difference between these $t0$ and $t1$ skill shares is calculated for all skills required by occupation o in either $t1$ or $t0$. The sum of these absolute skill share differences, SkillChange_o, captures the skill change for occupation o from $t0$ to $t1$.

To account for a secular increase in job post number and skill number per post,[2] Deming and Noray weight the skill change rate calculated from equation (1)a by multiplying the inverse growth rate of the average number of skills per job in an occupation. Specifically, this is measured as the ratio of skill occurrence divided by post number in 2007 to that in 2019, for each occupation (see equation (1)b). In equation (1)b, the numerator is the number of skills required for occupation o in $t0$ divided by its required skill number in $t1$. The denominator is the number of job posts of occupation o in $t0$ divided by its number of job posts in $t1$. This weight is not a correction against a principled null model, but simply weights occupations that grow in complexity less heavily than those that do not.

$$\text{SkillChange}_o = \sum_{s=1}^{S} \left\{ \text{Abs} \left[\left(\frac{\text{skill}_o^s}{\text{JobAds}_o} \right)_{t1} - \left(\frac{\text{skill}_o^s}{\text{JobAds}_o} \right)_{t0} \right] \right\} \tag{1)a}$$

$$\text{Weight}_o = \frac{\frac{\text{NSkill}_{o,t0}}{\text{NSkill}_{o,t1}}}{\frac{\text{NAds}_{o,t0}}{\text{NAds}_{o,t1}}} \tag{1)b}$$

We replicate this measurement on the same job ads dataset compiled by LightCast, limiting the sample to posts with non-missing employer and MSA information. Our replication for 6-digit SOC occupation skill change rates from 2007 to 2019 highly correlates with the scores listed in [2] appendix with Pearson correlation r=0.87 (p<0.001). The replicated measure does not obtain exactly the same values for two reasons: (1) We only have access to the first 5 month of job posts data in 2019, whereas [2] uses 10 months' data for 2019; (2) [2] further filter the sample by only retaining posts with employers that could be matched to a specific Compustat dataset inaccessible to us.

2.2 Occupational Skill Change Measurements in Figure 1c and d Illustration

In Fig. 1c, the area under the curve of each occupation corresponds to Deming and Noray's (2020)[2] job skill change measurement discussed in section 2.1. Note that all skills appearing in the job ads of a given occupation are taken into account. Because the measurement sums up the probability change for each skill required by the job in either start or ending year, we can easily decompose the total skill change for an occupation and rank skills by the proportion of occupation change for which they account. Fig. 1c presents the individual skill change for computer programer and food batchmaker in a ranked order from highest to the lowest.

For Fig. 1d, we first calculate the occupational skill vector change for Food Batchmaker and Computer Programmer using the method described in Methods section and applied to our main analyses. Namely, we represent the occupation skill content in 2010 and 2018 with the average vector of top 5% core skills required in those two years, respectively. With these occupation vector representations, we measure occupational skill change as one minus the cosine distance between occupation vectors in 2010 and 2018.

We then locate two groups of skills that contribute to the occupation skill change and attribute occupation level change to each skill. For each new core skill that emerges in 2018, we remove it and recalculate the occupation vector representation in 2018 based on the remaining skills. We then use the cosine distance between this adjusted 2018 occupation representation and the 2010 representation to estimate the occupational skill vector change rate that would have occurred if a given new skill were not added in 2018. We approximate the change that a given skill accounts for as the absolute value of the difference between the real occupation skill vector change rate and this controlled occupation skill vector change rate. Similarly, for each removed core skill that only appears in 2010, we estimate the adjusted occupation skill vector change rate that would have arisen if this skill were absent in 2010. Using the same approach, we then estimate the change contributed by each removed skill.

In Fig.1d, for each occupation, we rank all these new 2018 core skills and removed 2010 core skills by their estimated individual contribution to the occupation level change in skill embedding distance. With a comparable arrangement with Fig. 1c, the area under the curve for each occupation in Fig. 1d sums up these individual skill level contributions and approximates occupation level skill distance change. Note that because our measure for occupational skill change does not naturally decompose to individual skill level as Deming and Noray's (2020)[2] approach, the area under the curve estimated from Fig. 1d (0.066 for food batchmaker, 0.027 for computer programmer) is slightly different from the real occupational-level skill change calculated by our holistic approach and applied for all analyses (0.082 for food batchmaker, 0.017 for computer programmer). Therefore, we report the real occupational level change in Fig. 1d text annotation to stay consistent with the rest of the paper.

2.3 Cluster Approach to Measure Occupational Skill Change

In order to construct a more conservative test of our conclusion regarding the reskilling burden for lower-paid and lower-educated occupations relative to that of Deming and Noray[2], we develop a version of occupational skill formally equivalent to Deming and Noray's approach, but using data-driven skill clusters identified from the skill co-occurrence network rather than individual skills.

We used 2010 Lightcast job postings data to construct a skill co-occurrence network, a topological representation of skills linked by co-presence within job advertisements[3]. Our approach adds geometric precision to create a Pointwise Mutual Information (PMI) skill network. We first calculate the PMI of each pair of skills using equation (2). With each skill represented as a node in the network, an edge between two nodes is added if the PMI between the two skills is larger than 0, implying that these two skills are more likely to co-occur in the same or similar job posts than expected under independence[4]. The weight for each edge is the PMI score between the two skill nodes connected by the given edge. The 6 skill communities detected from the PMI network by the commonly-used Louvain community detection algorithm are: (a) business skills; (b) engineering, technical, and physical skills; (c) programming skills; (d) clerical and administrative skills; (e) scientific knowledge; (f) health and medical care skills. The modularity of the partition is 0.51, indicating that this division is very strong, and represents substantial community structure in the network[5].

For skill i and j,

$$\mathrm{PMI}_{i,j} = \log \frac{p_{i,j}}{p_i \times p_j} \tag{2}$$

where p refers to probability and

$$p_{i,j} = \sum_{v=1}^{V} p(i|v) \times p(j|v) \times p(v) \tag{3}$$

denotes the probability of skill i and j co-occuring in the same or a similar job post v.

Utilizing the 6 communities automatically detected from the PMI network, we calculated skill community change with equation (4). Fig. S2a shows that this measurement reverses the assessment of Deming and Noray[2]. Fig. S2b suggests that this measurement highly correlates with our main skill vector change measurement.

$$\text{SkillChange}_o = \sum_{sc=1}^{SC} \left\{ \text{Abs}\left[\left(\frac{\text{NSkill}_o^{sc}}{\text{NSkill}_o} \right)_{t1} - \left(\frac{\text{NSkill}_o^{sc}}{\text{NSkill}_o} \right)_{t0} \right] \right\} \tag{4}$$

2.4 Embedding Validation: Internal Validation

We created *t*-SNE visualization for the vectors of 6652 skills that have non-missing skill family labels classified by the data vendor Lightcast (Fig. S3). We color code the skills with skill family labels. Similarly, Fig. S4 is the t-SNE visualization for 6-digit SOC occupation vectors, color-coded with 2-digit SOC occupation group names. In both figures, skills and occupations belonging to the same broader group form meaningful clusters. Similar skill and occupation groups are also close to each other in the space. These visualizations demonstrate the effectiveness of the skill2vec space in capturing the relative distances between skills.

Furthermore, we compared occupational skill vectors with corresponding occupation title word vectors derived from the skill2vec space to assess if they are consistently closer to each other than with other random word vectors. As the skill2vec space consists of 15,182 skills in the Lightcast job postings data, many words in occupation titles do not exist in this corpus. Therefore, we focus on 132 occupations with parts of its title represented in the skill embedding space and calculate their corresponding title vectors as the average of the matched phrases vectors. As a baseline comparison, we generate an average random word vector for each occupation by randomly selecting the same number of phrases as the matched occupation title phrases from the skill embedding space. For example, two phrases associated with the occupation title Public Relations and Fundraising Managers are represented in the skill embedding space: public relations and fundraising. The title vector of this occupation is the average vector of these two phrases. And the random word vector is the average of two randomly selected phrases from the skill embedding space. We calculated the cosine similarities between average occupational skill vectors and their corresponding title vectors, then compared these values to the cosine similarities between average occupational skill vectors and random word vectors.

Fig. S5a plots the distribution of these two groups of cosine similarities for the 132 occupations. The former group is generally larger than the latter random baseline group. A two-sample *t*-Test shows that the mean of the similarity between occupation skill and title vectors is statistically significantly larger than the mean of the similarity between occupation skill and random word vectors (t-statistic=30.248, p<0.001). Fig. S5b is a scatter plot where the x-axis denotes the cosine similarity between an occupation's average skill vectors and average title vectors, whereas the y-axis represents the cosine similarity between the average skill vectors and average random word vectors. All occupation dots are below the diagonal line benchmarking the equivalence of the two values, confirming that occupational skill vectors are consistently closer to their corresponding occupation title word vectors than with random word vectors derived from the same embedding space.

2.5 Embedding Validation: Pre-trained LLM

As *skill2vec* is trained solely on job postings, it may skew towards overrepresented occupations and skills in the online job space. To address this potential bias, we validate our embedding space using a large language model pre-trained on a broader corpus of text data: *Labor Space*[6]. *Labor Space* is derived from Google's BERT, which is trained on Wikipedia and the Google Books Corpus. Kim et al.[6] fine tuned BERT with representative descriptions of different levels of labor market entities from various corpora, including Occupational Information Network (O*NET) and European Skills, Competences, Qualifications, and Occupations (ESCO). *Labor Space* therefore captures the semantic distances between labor market's key elements.

We focus on 259 occupations and 7,219 skills in our data, as each token within these has an identical match in the *Labor Space*. We calculated the pairwise cosine similarity between all occupation pairs and skill pairs using the *Labor Space* word embedding representation and our *skill2vec* skill embedding representation, respectively. We then compared the two sets of pairwise cosine similarities obtained from the two spaces. For occupation pairs, the Pearson correlation between the two sets of pairwise cosine similarities is 0.34 (p <0.001). For skill pairs, the Pearson correlation between the two sets of pairwise cosine similarities is 0.29 (p <0.001). We also generated a random baseline reference by randomly reshuffling occupation and skill names in LABERT vectors before calculating correlations. The random baseline correlations are 0.00 (p = 0.89) for occupation pairs and 0.00 (p = 0.36) for skill pairs. This analysis suggests that the relative distances between skills and occupations in the skill2vec space are consistent with that encoded in the LABERT space.

2.6 Embedding Validation: Factor Analysis

In this section, we compare the skill embedding approach to the more traditional factor analysis approach used in labor economics work to derive occupational distance measures[7,8]. To identify latent skill factors, we use 2018 job postings data to construct an occupation-skill probability matrix. Each occupation-skill cell consists of the number of job posts for the occupation requiring the given skill divided by the number of all job posts for the occupation. To generate a larger training dataset while maintaining model runtime efficiency, each occupation is weighted by either 0.1% of its total job post number or 1, whichever is greater. This weighting approach results in a dataset of 28,068 rows, providing a larger sample size than previously used for identifying skill factors in other works. To prepare for factor analysis, we first remove extremely low-variance skill columns and skills that are perfectly or almost perfectly correlated with other skills in the matrix. This preprocessing reduces skill variables from around 15000 to 1554. We then standardize the matrix to have a mean of 0 and a standard deviation of 1.

Next, we apply factor analysis to identify 200 orthogonal skill factors. Together, these factors account for 88% of the variance in the occupational skill probability matrix. We transform the occupational skill probability matrix with the factor loadings to represent each occupation as a vector of 200 factor scores. With these 200-dimensional occupation factor vectors, we calculate the distances between all pairs of occupations and compare them with the pairwise vector distances calculated from skill embedding representation of occupations. The Euclidean distance or cosine similarity between occupation pairs represented by factors post moderate, statistically significant correlations with the distances calculated based on skill embedding representation of

occupations: the pearson correlation is 0.52 for Euclidean distance and 0.59 for cosine similarity (both $p < 0.001$).

The two approaches also perform similarly in predicting job switches based on 2018 CPS data, with skill2vec explaining slightly more variance in job switches. We regress worker moves between a pair of occupations on the pairwise occupation distances and a baseline prediction based on occupation popularity (i.e., the logarithm of the product of employment in the two occupations using 2018 BLS data). We found that skill2vec-based distances explain more variance in job transitions than factor-based distances, regardless of how distance is defined. With factor-based occupation-pair distances, the R-squared in the regression is 0.095 for Euclidean distance, and 0.137 for cosine similarity. When pairwise occupation distances are measured with skill embedding approach, the R-squared in the regression is 0.131 (37% increase) for Euclidean distance, and 0.141 (3% increase) for cosines similarity.

2.7 Identifying the Direction of Occupational Skill Change with Skill Atoms

Occupation vectors predict not only the magnitude of skill change but also its direction. First, we construct a "coordinate system" of the skill space by using the discourse atom topic modeling approach, which performs k-SVD matrix factorization on skill vectors to accurately and efficiently label the skill space[9,10]. The derived vectors or "skill atoms"[9] represent near-orthogonal axes capturing the essential "bases" of distinct human capacity, which can be linearly combined to recover the vector representations of 15,182 actual skills. Specifically, each skill is represented as a linear combination of k skill atoms. We trained models by setting the atom number k from 50 to 500. The model performs best with 210 atoms based on a balance between (1) R^2, which measures how well the atoms predict all skill vectors; and (2) topic diversity, which measures how distinct the atoms are from one another.

After we obtain the 210 skill atoms to anchor our skill-space as coordinates, we specify the direction of occupational skill change within this system. We quantify the rise and fall of skill atoms as a function of how all 721 occupations shift collectively. Rising atoms are those a majority of occupations approach, and declining atoms are those a majority of occupations depart. Specifically, we calculate the overall importance of a skill atom as the sum of its weights across all occupations in that year and compare how overall importance changed between 2010 and 2018 following the procedures detailed below.

1) The compositionality of occupations based on skill atoms:
    a) Map each occupation to 5% core skills filtered by skill probability
    b) Map each skill to atoms - Denote each skill s with its weights on each atom j:

$$s = \sum_{j=1}^{210} \text{weight}_{sj} \tag{5}$$

    Note that according to the current model, there are only 5 non-zero $weight_{sj}$ for each skill s.
    c) Map each occupation to atoms - Denote each of the 721 occupations in a given year (2010 or 2018) as a combination of atoms by adding up its core skills

represented by atoms in the equation (6). The weight of each atom for each occupation is normalized by dividing the sum of all atom weights for the given occupation. For an occupation L_t with S_t core skills at time point *t*:

$$L_t = \sum_{j=1}^{210} \text{weight}_{otj} \tag{6}$$

in which

$$\text{weight}_{otj} = \frac{\sum_{s=1}^{st} \text{weight}_{sj}}{\sum_{j=1}^{210} \sum_{s=1}^{st} \text{weight}_{sj}} \tag{7}$$

2) The overall importance change of skill atoms on the job space:
   a) Measure the overall importance level for each skill atom on the job space at a given time point - for each atom *j* at each time point *t*, summing up its normalized weight for each occupation *o* (weight_{otj}):

   $$\text{Importance}_{jt} = \sum_{o=1}^{727} \text{weight}_{otj} \tag{8}$$

   b) Measure atom overall importance change on the job space between 2010 and 2018 - for atom *j*:

   $$\text{ImportanceChange}_j = \text{Importance}_{j2018} - \text{Importance}_{j2010} \tag{9}$$

Fig.S6a presents these 210 skill atoms in a matrix of 14 rows and 15 columns. To demonstrate the relative location of all 210 atoms on a 2-D graph, we first apply the T-SNE transformation on the original skill atom vectors to reduce their dimension from 200 to 2. We then construct a grid of 15 columns and 14 rows on the area between the lowest and highest values for each of the 2 dimensions for all atoms. Finally, for each node in the grid, we assign the nearest unassigned atom to occupy it. Finally, we employed two human coders to label these 210 skill atoms “human”" or “machine”-related based on the closest 25 skills to a given skill atom in the space to observe how these two kinds of skill atoms rose or declined in the past decade. Fig.S6b shows two exemplary occupations and their most dramatically altered atoms, 2010 to 2018. We present skill atoms that decline in importance across all jobs and atoms that increase in importance in this period (Fig.S6c-d) to highlight global transformations of skill in the U.S. labor market. Future work can build on these spaces to explore other causes and consequences of the rise and fall of skill atoms for skill change across distinct occupations.

3. Supplementary Note 3

3.1 Job Zone and Skill Change

We use O*NET five-level job zone classification (i.e., “little or no preparation”, “some preparation”, “medium preparation,” “considerable preparation”, “extensive preparation”) as a

proxy for occupational skill level. The job zone measure (https://www.onetonline.org/help/online/zones) reflects on-the-job training and experience in addition to formal education in capturing the first-order learning costs associated with each job—their relative difference from no education. Fig. S7 demonstrates that skill change decreases as job zones increase from 1 to 5 except that jobs in zone 5 change more than those in zone 4.

3.2 Occupational Skill Change as the Most Significant Skill Transition

In the main paper, we measure occupational skill change as the distance between the occupational average skill vector at different time points. This approach may underestimate the skill change of higher-skilled occupations compared to lower-skilled occupations under a situation when both groups of occupations add the same number of distant new skills, and the higher-skilled occupations also add many more similar new skills. With this skill-adding structure, the higher-skilled occupations change more than the lower-skilled occupations, yet the large number of similar skill additions may end up "diluting" the significant change embodied in the addition of dissimilar skills. To address this potential bias, we developed an alternative measurement to characterize occupational skill change as its most significant distance of skill transition, and tested whether lower-skilled occupations still change more than higher-skilled counterparts.

For each occupation o, we identify the n newly-added core skills in 2018. For each of these new skills, s_n, we find its nearest skill (based on embedding distance), s_{n10}, among the 2010 core skills of o. We consider the embedding distance between s_n and s_{n10} as reflecting the minimum amount of skill transition needed for a worker who held o in 2010 to acquire s_n. Calculating such distance from all pairs of s_n and s_{n10}, we use the maximum pairwise skill transition distance to measure the most significant individual skill transition a worker who held o in 2010 needs to experience to keep their job in 2018. To rule out the influence of outliers that may arise at the maximum boundary, we also investigated measurements based on various top positions within the distribution of individual skill transition. Specifically, we considered the pairwise skill transition distance that ranks the top 5% and 10% when ordered from the largest to the smallest.

In Table S1, we regress these three measurements of occupational skill change on occupational skill complexity (natural log of average core skill number), natural log of average occupational annual wage, and average education year, respectively. Results show that lower-skilled occupations still have larger skill change than higher-skilled occupations when skill change is measured as the most significant individual skill transition.

3.3 Upskilling of Low-Skilled Jobs

To understand whether the significant skill change that took place in low-skilled jobs denoted an upskilling direction, we calculated the skill gap between the low-skilled job group and high-skilled job group in 2010 and 2018 and examined whether the gap has narrowed over the years. In Fig. S8a-c, we divided occupations into two groups based on three different criteria: (a) occupations with core skill number at or above median core skill number across all occupations are classified into the high-skilled group, and the rest is classified into the low-skilled groups; (b) occupations with average annual pay at or above the median across all occupations are classified

into the high-skilled group, and the rest is classified into the low-skilled groups; (c) occupations with college or above education requirement are classified into the high-skilled group, and the rest is classified into the low-skilled groups.

We calculate the skill gap between the high-skilled and low-skilled occupation group in a given year by pairing nearest occupations across the two groups and average the pairwise distance. Specifically, for each occupation in the high-skilled group in 2010, we pair it with its nearest occupation (based on 2010 average skill vector) among all low-skilled group occupations in 2010. Following the same procedure, we find the nearest occupation in the high-skilled group for each low-skilled group occupation in 2010. The skill distance between the high-skilled and low-skilled occupation groups in 2010 is measured as the average of all these pairwise occupation skill vector distances in 2010. The left yellow bars in Fig.S8a-c represent the skill distance between the high-skilled and low-skilled occupation groups in 2010, whereas the right green bars in Fig.S8a-c represent the inter-group skill distance in 2018, calculated based on the same approach described above. The three panels all suggest an 'upskilling' story of low-skilled occupations moving closer to high-skilled occupations in terms of skill requirements from 2010 to 2018. These patterns are statistically significant with two-tailed tests of mean difference between 2010 and 2018 inter-group skill distance (for Fig. S8a, $t(720) = 20.50$ with two-tailed t test of mean difference, $p < 0.001$, Cohen's $d = 0.76$, 95% CI [0.020, 0.025]; for Fig. S8b, $t(720) = 15.81$ with two-tailed test of mean difference, $p < 0.001$, Cohen's $d = 0.59$, 95% CI [0.017, 0.022]; for Fig. S8c, $t(720) = 14.37$ with two-tailed t test of mean difference, $p < 0.001$, Cohen's $d = 0.54$, 95% CI [0.017, 0.023]).

3.4 Meaning and Significance of Occupational Skill Change: Re-Educational Costs

We further explore the meaning and significance of occupational skill change by demonstrating that distance in the skill space correlates with the amount of re-education required for workers to move from one distribution of skills to another. To draw comparison between jobs with marginal skill differences, we undertake the following analysis:

1. We randomly select a 10% sample from all 2010 job posts, and identify among them pairs of job posts, x and y, from the same occupation, where post y encompasses all skills listed in post x, but also possesses additional skills. Table S2 presents two job ad examples picked from January 2010 data to illustrate the type of comparison we are making in calculating the extra education necessitated by marginal skill change. The first example includes two ads for administrative assistant positions at entry and senior levels. Both ads require basic administrative skills, such as communication and administrative support, as well as proficiency in Microsoft Office tools like Excel, Word, and PowerPoint. The senior position, however, also requires specialized skills in customer management, such as Siebel CRM, Portal Tools, Customer Information Control System (CICS), Direct Mail, and Newsletters. Here, the skill distance between the two ads is 0.17, and the senior role requires an additional 2 years of education compared to the entry-level role (16 vs 14 years). The second example includes two job ads for registered nurses with different levels of specializations. Both positions require basic patient care and service skills, whereas the position that specializes in Interventional Radiology additionally requires more advanced skills on this direction, such as Advanced Cardiac Life Support (ACLS), Catheterization, Catheterization Laboratory (CATH LAB), Critical

Care, and Interventional Radiology (IR). The skill distance between the two job posts is 0.15 and the education year difference is 2 years.

2. Using the sample selected in step 1, we further select pairs where *y* requires more education years than *x*.
3. Using this subsample, we plot the distribution of the number of additional skills in *y* compared with *x* (which typically follows a unimodal distribution, see Fig. S9), and identify the peak of that distribution m_o. This peak is more likely than other numbers in the distribution to correspond to a regularly-added combination of skills, typically obtained through more advanced education. Fig. S9 illustrates eight example occupations, each showcasing distinct distribution types peaking at varying numbers of additional skills.
4. Next, we analyze a subset of 6,585,524 job post pairs from the Step 1 sample where *x* and *y* belong to the same occupation *o*, and *y* requires m_o, m_o-1, or m_o+1 more skills beyond the common skill set shared with *x*. Because our data do not allow a comprehensive measurement of all sorts of re-training for skill acquisition and there are only a few levels of education degrees, we apply step 4 to upweight cases where formal education requirements differ between job posts within the same occupation. Note that in the analytical sample built in this step, job post *y* may add m_o skills but does not require a higher degree than *x* because either those skills are not necessarily the regularly-added combination of skills, or could be obtained without a new formal degree. We aim to test whether larger skill distances predict degree requirement changes and more substantial education year differences by comparing a range of cases, encompassing those with no meaningful degree changes, those with such changes, and variations in the magnitude of change between them. In the first column of Table S3, we regress the pairwise education year difference on the embedding distance between the average skill vectors of the jobs. These variables show a positive and statistically significant relationship.
5. Because job posts pairs within each occupation tend to present similar pattern, we aggregate the data to the occupation level by calculating for each the average required education year difference and skill distance based on three groups of job posts pairs: pairs with m_o, m_o*-1,* or m_o*+1* skill additions, respectively, resulting in 1257 data points.

Fig. S10 is based on this aggregated analysis and shows that marginal skill distance predicts educational difference. The 2nd column in Table S3 presents the regression at occupation level and shows a stronger positive correlation between skill distance and education requirement difference. We then transform skill distances into z-scores in the 3rd column of Table S3 to facilitate the interpretation of results. We find that one standard deviation increase in skill distance is associated with 0.172 years more schooling.

To ensure these patterns are not a production of this specific sample, we applied different random selection approaches to draw samples from 2010 data, including with different seeds or randomly select a full month data. Based on the six different random samples we examined, the association between education year difference and skill distance is always positive and statistically significant. The average of the Pearson correlation of these variables is 0.05 with raw job post unit of analysis, and 0.14 with aggregated occupation level of analysis. The average coefficient from regressing education year difference on the z-score of skill distance is 0.1 at raw job post

level, and 0.17 at aggregated occupation level. We note that this measured shift in educational *level* does not account for the necessary shift in educational *type*. For example, shifting from a data analyst job in a media company to that in a biotech company may not require a higher level of education (e.g., a Bachelor degree), but is more likely to require a degree with a different major as distance in the skill space increases.

3.5 Labor Market Tightness and Skill Change

We computed the year-on-year average occupational skill changes (e.g., 2010-2011, 2011-2012, etc.) across all occupations from 2010-2018 and found a positive correlation with the yearly civilian unemployment rate released by BLS: pearson coefficient 0.95 with p-value less than 0.005. Fig. S11 shows that the yearly average occupational skill changes decline with the yearly unemployment rate from 2010-2018. Given that our skill change measure largely captures upskilling directions, this pattern is consistent with the business cycle literature [11–13]. As the labor market becomes tighter from 2010 to 2018, employers have gradually eased off their upskilling attempts in skill requirements as they decrease educational attainment and experience requirements.

3.6 Robustness Check: Controlling for Employer Concentration, Within-Occupation Job Role Homogeneity, and its Changes

This and the following section present regression-based robustness checks for the two findings that (1) lower skilled occupations experience more skill changes than higher-skilled occupations; and (2) larger firms and markets experience less skill changes than their smaller counterparts. The regressions used to verify the first finding use occupation as the unit of analysis and weight each occupation by the average number of job posts in 2010 and 2018 to adjust for the fact that some occupation cells are larger and have more accurate averages. To verify the second finding, we ran two sets of regressions with different analytical units. The first one uses the firm-commuting zone(cz)-occupation as an analytical unit to track within firm-region-occupation changes. Each firm-cz-occupation unit is weighted by the corresponding average number of job posts in 2010 and 2018. Because this analysis could only be performed on the subsample of units that appeared in both 2010 and 2018, we ran the second set of regressions to include occupations that disappeared or newly appeared in 2018. In the second set of regressions, the analytical units are firm-cz that appeared in both 2010 and 2018. Each firm-cz is weighted by the corresponding average number of job posts in 2010 and 2018.

The regressions in this section address several potential biases. First, for occupations with high average local employer concentration, their skill change could be overwhelmingly determined by changes driven by a few employers. Therefore, we control for employer concentration in regressions to ensure that the variation in job skill change by job skill complexity, employer size and local market size does not merely reflect variation in occupational employer concentration within the local labor market.

Following[14–16], we measure employer concentration with the Herfindahl-Hirschman Index (HHI) of the share of Lightcast vacancy postings from each employer for each SOC 6-digit occupation within a local labor market in a given year, as specified in equation (10). To align with the geographical unit used in examining job skill variation by local labor market size, here we identify the local labor market as commuting zones.

$$\text{EmpConcentr}_{o,k,t} = \sum_{i=1}^{N} \left(\frac{\text{JobAds}_{i,o,k,t}}{\sum_{i=1}^{N} \text{JobAds}_{i,o,k,t}} \right)^2 \tag{10}$$

where $JobAds_{i,o,k,t}$ refers to the number of Lightcast vacancy advertisements posted by employer i on occupation o in local market k during year t. In the occupation unit regressions, we aggregate this measure to occupation level as average occupation employer concentration across all local markets, year 2010 and 2018. In the firm-cz-occupation unit regressions, we take the 2010 and 2018 averages for employer concentration at occupation-cz level. And for firm-cz unit regressions, employer concentration is aggregated to cz level, averaging across all occupations in year 2010 and 2018.

Another potential bias lies in the measurement of occupational skill change: it reflects both shifts in within-job-role skill requirements and job role composition within an occupation. A 6-digit SOC occupation is a meaningful unit of analysis for our purpose because it is a relatively consistent framework for analyzing individuals' roles in the labor market over time. Based on 2018 CPS-ASEC data, approximately 87% of observations (with non-missing occupation variables) stay in the same occupation as the prior year. Workers in the same occupations tend to share similar human capital, job task requirements, working conditions, and career trajectories. However, skill requirements do vary within occupation and across but sometimes also within firms and regions. An occupation can encompass multiple closely connected but not completely identical sub-occupational job roles. When some of the sub-occupational job roles look too different from others, it may raise the concern that they are misclassified. While job role composition change is also an important part of occupational skill change, we want to ensure that it is not driving the results, especially when potentially misclassified job roles blend in.

Therefore, we construct a measure for occupational job role composition change from 2010 to 2018 with changes in within-occupational job role homogeneity level. With each job post represented as the average required skill vectors, an occupation's job role homogeneity is measured by the average pairwise job post similarity for all job posts of the given occupation in a given year. A higher score means less within-occupation variance: a score of 1 means all job posts are identical in skill contents. Changes in this metric reflect whether an occupation becomes more or less diverse in its job role composition by adding or abandoning job roles that are very different from the main job roles. To single out skill changes from job role composition changes, we control for within-occupation job role homogeneity level change in all the regressions. Additionally, occupations internally more homogeneous might be in the more stable job fields, embed in more specific contexts, and therefore become prone to have less dramatic skill changes. Therefore, we also control for occupational job role homogeneity to rule out any potential confounding effect.

For occupation unit regressions, we randomly sampled 5% job posts for each occupation in a given year to construct the within-occupation job role homogeneity measure. The average occupational homogeneity score based on 2010 and 2018 data is 0.63. For firm-cz-occupation unit regressions, we use all job posts for each unit to measure within firm-cz-occupation job role homogeneity. The average firm-cz-occupation homogeneity score based on 2010 and 2018 data

is 0.95. For firm-cz unit regressions, we take the average of within firm-cz-occupation job role homogeneity for each firm-cz unit.

Table S4 presents OLS regression results explaining job skill change variation with the three different measurements for job skill level: job skill complexity, pay, and education. Consistent with Fig. 2 in the main body, models 1-3 show that lower-skilled occupations change more. Models 4-5 demonstrate that this pattern still holds when employer concentration, within-occupation job role homogeneity level and its temporal change are controlled. The coefficients for employer concentration align with the findings in Hershbein et al[16]—occupations with larger local employer concentration experience more re-skilling.

Table S5 presents a series of occupational fixed effect model results describing how skill content change for the same occupation varies with organization and local market size, based on the sample of firm-cz-occupations that appear in both 2010 and 2018. The occupation fixed effect controls for time-invariant occupational characteristics, including within-occupational job role homogeneity level. Model 1 and 2 show that larger firms and larger local markets experience smaller skill changes for the same occupation than their smaller counterparts. As shown in model 3-4, these results are still robust when employer concentration and job role composition change is taken into consideration. Surprisingly, when employer and/or labor market size is controlled, larger employer concentration correlates with smaller job skill change. Given that employer concentration is constructed at a relatively narrow region level, this pattern might result from the high collinearity between local market size and employer concentration. In model 5, when we put both employer and market size together in the same model with all controls, the coefficient of employer size is no longer statistically significant (p=0.065). This suggests that employer size and market size are correlated and that the latter has a more robust and statistically independent association with the within firm-cz-occupation skill change.

While table S5 focuses on within firm-cz-occupation skill changes, Table S6 examines firm-cz level overall skill changes that incorporate disappearing and newly-appearing occupations in 2018. The weighted firm-cz unit regressions show that larger firms and local markets have smaller overall skill changes. Coefficients for firm size and market size in Table S6 are smaller than their coefficients in Table S5, suggesting that variations in skill changes by entity sizes are slightly less pronounced for overall entity skill changes than entity-occupation skill changes. Such differences could be due to the different samples involved or that larger firms and markets include more rapidly-changing occupations.

3.7 Robustness Check: Different Job Content Scope with Skill Weights

While the Lightcast job postings data provide rich and valuable information on dynamic job skill requirements that enables analyses in this paper, the data also create the new task of identifying the relative importance of skills to jobs as compared with the O*NET data. In the main analysis, we adopt a discrete approach to account for skill importance differences in representing occupational skill content—limiting occupational skill composition to the most common 5% skills for a given occupation. This 5% threshold is arbitrary, however, and a discrete approach is not necessarily better than a continuous approach that assigns weights to each skill. Therefore, we present a series of robustness checks here with different job content scopes (all skills, top 50% core skills, and top 25% core skills) using a continuous approach to incorporate skill

importance differences: we represent occupation vectors as the sum of weighted skill vectors, as shown in equation (11).

$$\text{Vector}_{o,t} = \sum_{s=1}^{S_t} \frac{\text{Freq}_{o,s,t}}{\text{Freq}_{o,t}} \times \text{Vector}_s \tag{11}$$

where the weight is the proportion of occurrences of skill *s* in job posts on occupation *o* in year *t* among the sum of occurrences for all skills that appear in job posts on occupation *o* in year *t*.

Table S7 consists of three sets of estimations of model 4 in the main table S4 that explain job skill change variation with skill complexity using all skills, top 50% core skills, and 25% core skills to define job skill content, respectively. Similarly, Table S8 and 9 present estimations of model 5 from Table S5 and S6, respectively, testing the variation of skill change by organization and local market size with different job content scopes. Note that tables S8 and S9 have more observations than tables S5 and S6 because the main 5% core skill change approach is only applied to units (firm-cz-occupation or firm-cz) with at least 5 core skills in both 2010 and 2018. The current continuous skill change measures loosen the criteria to 5 total skills in both 2010 and 2018 and therefore largely expands the sample, especially after weighting. All findings are robust to the continuous skill weight approach and different thresholds for job skill content definition.

4. Supplementary Figures

4.1 Figure S1.Validation of Lightcast data representativeness

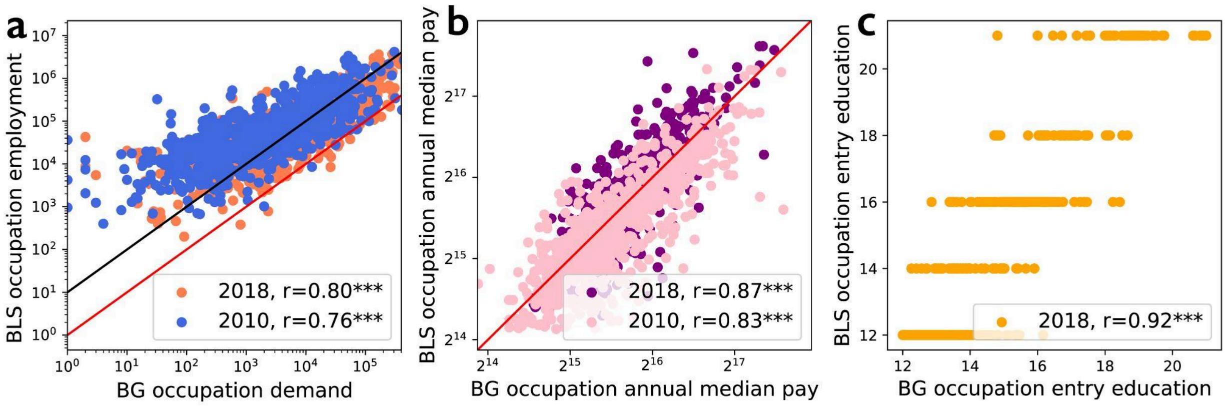

Supplementary Figure 1. Validation of Lightcast data representativeness: a, Lightcast occupational demand is highly consistent with the state of occupational employment in the U.S according to BLS data. Coral dots are overlapping occupations from the two data sources in 2018 (n=786 occupations), with x-axis showing Lightcast occupational demand and y-axis showing BLS occupational employment (pearson correlation r=0.80, p<0.001). Royal blue dots are overlapping occupations from the two data sources in 2010 (n=777 occupations), with x-axis showing Lightcast occupational demand and y-axis showing BLS occupational employment (pearson correlation r=0.76, p<0.001). The red line is the diagonal $y = x$. The black line shows the red line's vertical translation upward. **b,** Lightcast occupational median pay accurately represents occupational median pay in the U.S. Purple dots are occupations with salary data from both data sources in 2018 (n=772 occupations), with x-axis showing Lightcast occupational median annual salary and y-axis showing BLS occupational annual median salary (pearson correlation r=0.87, p<0.001). Pink dots are occupations with salary data from both data sources in 2010 (n=761 occupations), with x-axis showing Lightcast occupational median annual salary and y-axis showing BLS occupational annual median salary (pearson correlation r=0.83, p<0.001). The red line is the diagonal $y = x$. **c**, The Lightcast occupational entry education level information could be trusted to represent the occupational entry education requirement in the U.S. Orange dots represent occupations with entry education information from both data sources in 2018 (n=682 occupations), with x-axis showing Lightcast occupational average required entry education attainment years and y-axis showing BLS occupational average entry education attainment years (pearson correlation r=0.92, p<0.001).

4.2 Figure S2. Cluster approach to measure occupational skill change

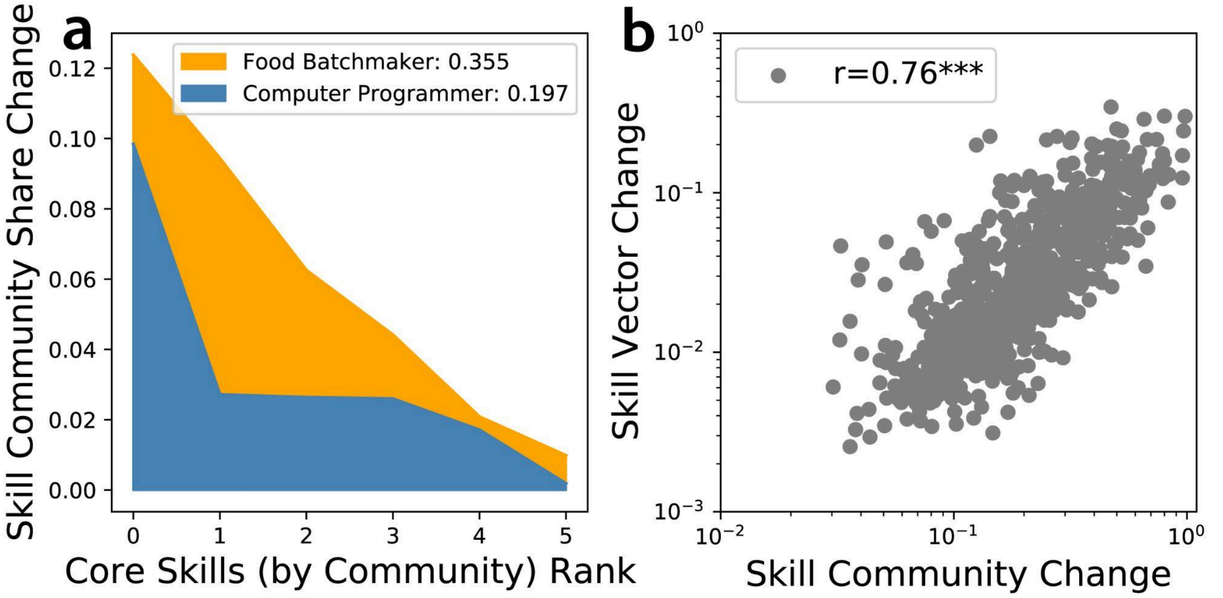

Supplementary Figure 2. Cluster approach to measure occupational skill change: a, After roughly controlling for skill distance through tracking skill community share change instead of individual skill probability change, food batchmakers are associated with larger skill change than programmers. Each unit of the *x* axis corresponds to one of the 6 skill communities ranked from highest to lowest in terms of skill community share change. The *y* axis denotes skill community share change for each skill community for Food Batch Maker (orange curve) and Computer Programmer (blue curve). Area under the curve (AUC) of skill community share change for each occupation demonstrates the sum of skill community changes for all 6 skill communities from 2010 to 2018: 0.355 for Food Batchmakers and 0.197 for Computer Programmers. **b**, Occupation skill change measured by skill community share change and skill vector change highly correlate (Pearson correlation r=0.76, p<0.001). Each grey dot is an occupation (n=721 occupations).

4.3 Figure S3. T-SNE visualization of skill vectors

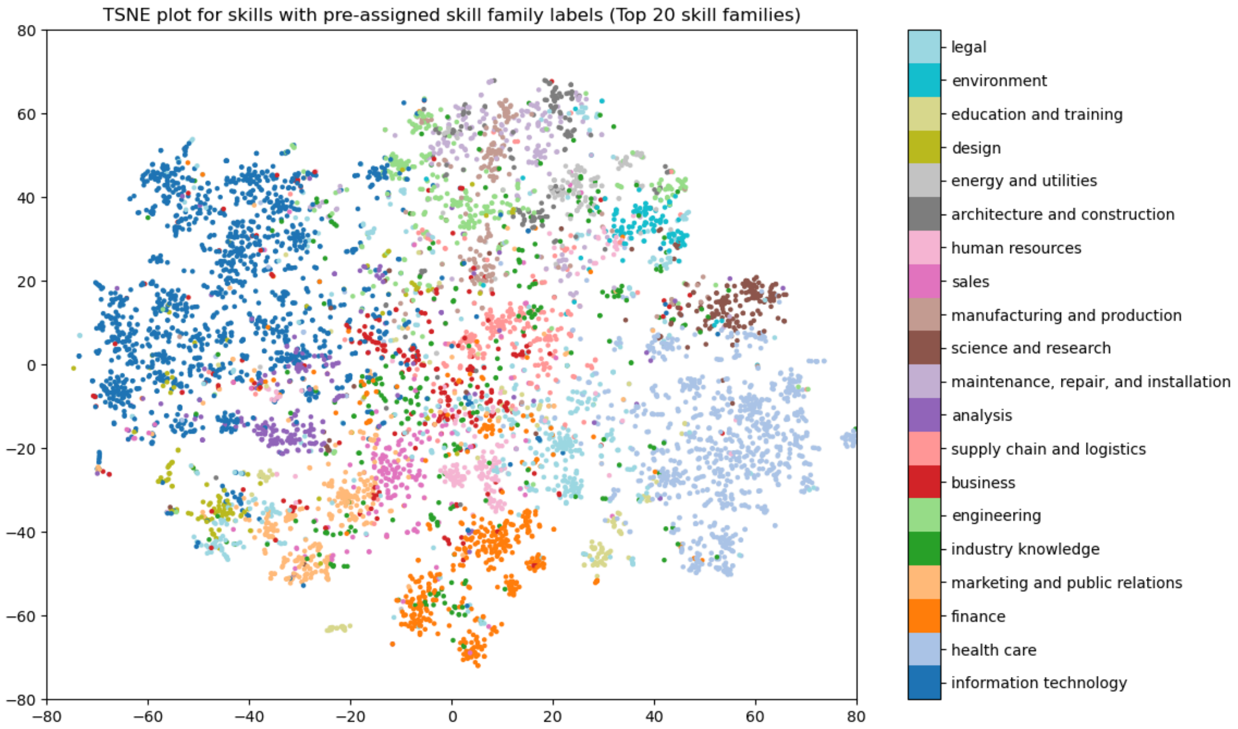

Supplementary Figure 3. T-SNE visualization of skill vectors: Each dot corresponds to the t-SNE representation of skill vectors for 6652 skills with 28 skill family labels classified by the data vendor lightcast. The skills are color-coded with skill family labels (only the most frequently appeared 20 labels are presented).

4.4 Figure S4. T-SNE visualization of occupation vectors

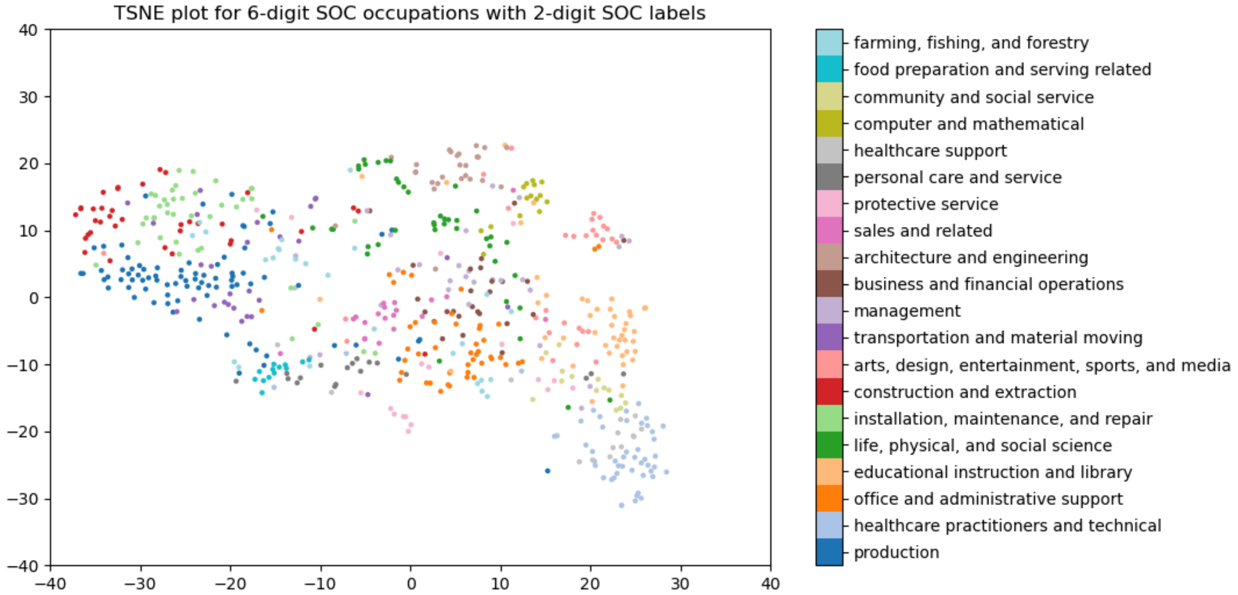

Supplementary Figure 4. T-SNE visualization of occupation vectors: Each dot corresponds to the t-SNE representation of 6-digit SOC occupation vectors (n=616 occupations) color-coded with 2-digit SOC occupation group names (only the most frequently appeared 20 groups among the 23 groups are presented).

4.5 Figure S5 Occupation skill vectors and title word vectors comparison

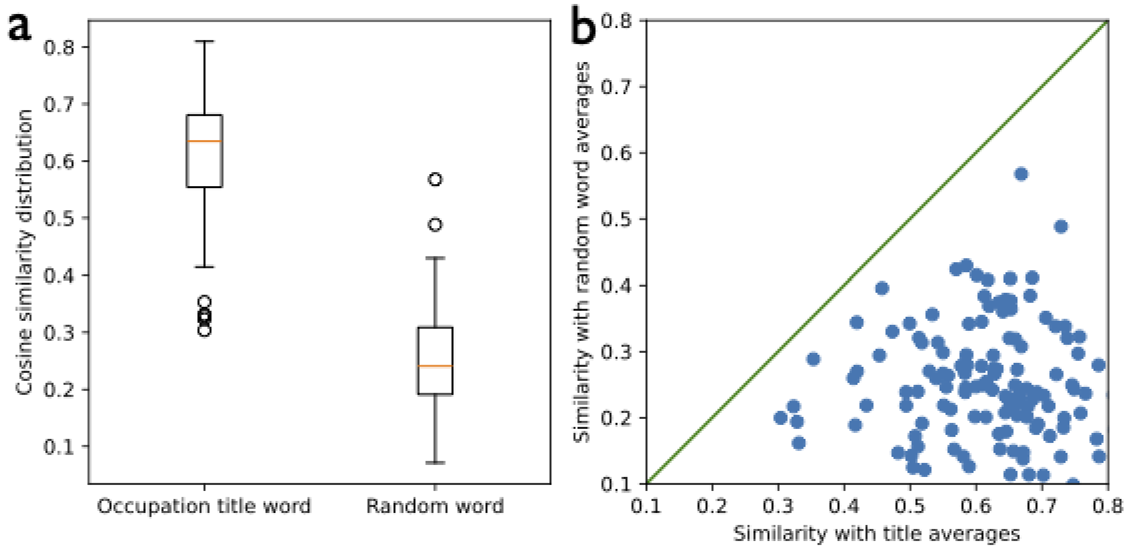

Supplementary Figure 5. Occupation skill vectors and title word vectors comparison: a, The box plots presents the distribution of cosine similarities between occupational average skill vectors and corresponding occupation title word vectors (left), and between occupational average skill vectors and random word vectors (right), for 132 occupations. In each box plot, the center line indicates the median, the box represents the interquartile range (25th–75th percentiles), the whiskers extend to 1.5 times this range, and points beyond the whiskers are plotted as outliers. **b,** Each dot represents an occupation (n=132), with x-axis denoting the cosine similarity between the occupation's average skill vector and its average title word vector, whereas y-axis corresponding to the cosine similarity between the occupation's average skill vector and average random words vector.

4.6 Figure S6. Occupations' re-skilling direction illustrated by skill atoms

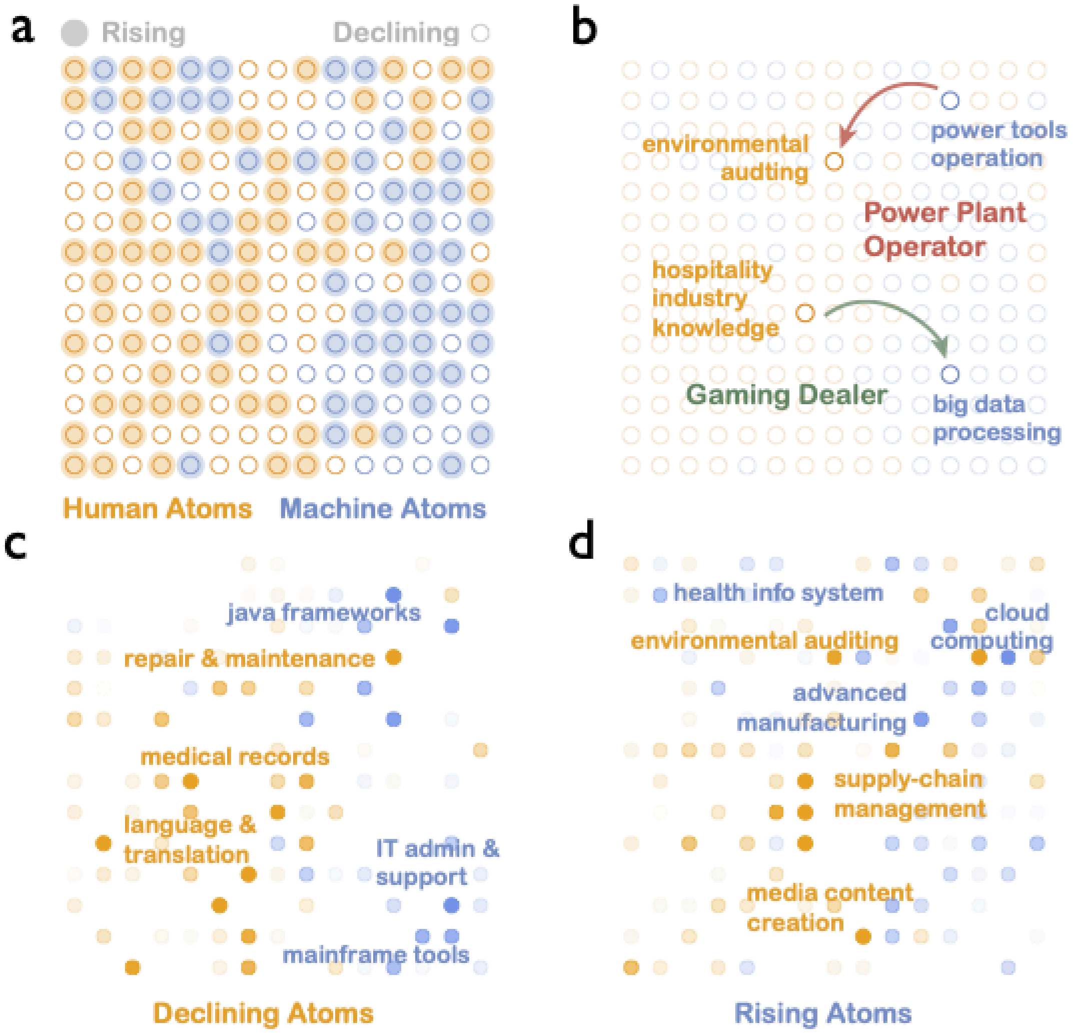

Supplementary Figure 6: Occupation re-skilling direction illustrated with skill atoms. a, Each dot denotes a latent skill atom (n=210) learned from the skill embedding space, embodying a meaningful dimension in the skill space and embedding its relationship to other skill atoms as well as to each specific skill. Orange skill atoms are defined predominantly by the human interface, and blue atoms are defined by requiring machine-operation and/or interface. Filled dots are skill atoms with rising importance on the skill demand space between 2010 and 2018, whereas the empty dots represent declining skill atoms. **b**, Examples of the re-skilling direction for individual occupations. For Power Plant Operators, the power tools operation atom declines the most and the environmental auditing atom rises most in importance (2010-2018). For Gaming Dealers employed by casinos, the skill atom that declines most in importance from 2010 to 2018 is hospitality industry knowledge, whereas the one that increases in importance most is big data processing. **c**, Declining skill atoms with negative (<0) importance change in the job space: the less transparent an atom, the larger its importance declines. **d**, Rising skill atoms with positive (>0) importance increase: the less transparent an atom, the larger its importance increases.

4.7 Figure S7. Job zone and skill change

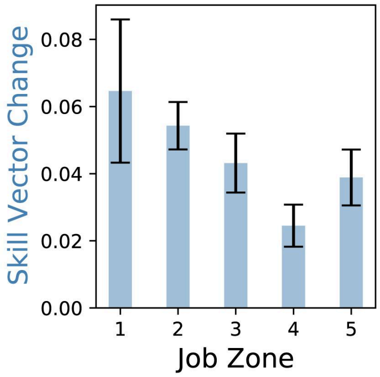

Supplementary Figure 7. Job zone and skill change: Occupations have higher average skill content change as job zone increase from 1-4. Each bar denotes the average skill vector change for occupations in a specific job zone labeled on the *x*-axis (n=602 occupations). Bars represent group mean, and error bars indicate the 95% confidence interval estimated via bootstrapping (50 resamples, each sized at 80% of the original data).

4.8 Figure S8. Upskilling of low-skilled jobs towards high-skilled jobs

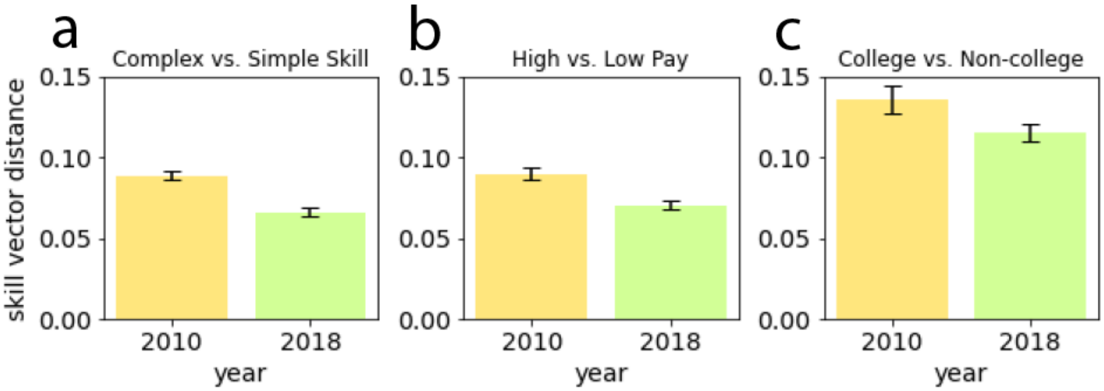

Supplementary Figure 8. Upskilling of low-skilled jobs towards high-skilled jobs. a. The skill distance between occupations with relatively complex and simple skills have narrowed from 2010 to 2018 (t(720) = 20.50 with two-tailed test of mean difference, $p < 0.001$, Cohen's d = 0.76, 95% CI [0.020, 0.025]). The left yellow bar represents the average skill vector distance between the occupation group with more complex skill requirements (at or above median core skill number across all occupations) and the group of occupations with simpler skill requirements in 2010 (n=721 occupations). The right green bar represents such distance in 2018 (n=721 occupations). In subplots a-c, all bars represent group mean, and error bars indicate the 95% confidence interval estimated via bootstrapping (50 resamples, each sized at 80% of the original data). **b.** The skill distance between occupations with relatively higher and lower pay have narrowed from 2010 to 2018 (t(720) = 15.81, two-tailed test of mean difference, $p < 0.001$, Cohen's d = 0.59, 95% CI [0.017, 0.022]). The left yellow bar represents the average skill vector distance between the occupation group with higher pay (at or above median in the distribution of annual median salary across all occupations) and the group of occupations with lower pay in 2010 (n=721 occupations). The right green bar represents such distance in 2018 (n=721 occupations). **c.** The skill distance between occupations with college and above vs. non-college degree requirements have narrowed from 2010 to 2018 (t(720) = 14.37, two-tailed test of mean difference, $p < 0.001$, Cohen's d = 0.54, 95% CI [0.017, 0.023]). The left yellow bar represents the average skill vector distance between the occupation group with college and above education requirement and the group of occupations with non-college degree requirements in 2010 (n=721 occupations). The right green bar represents such distance in 2018 (n=721 occupations).

4.9 Figure S9. Distribution of number of added skills by occupation

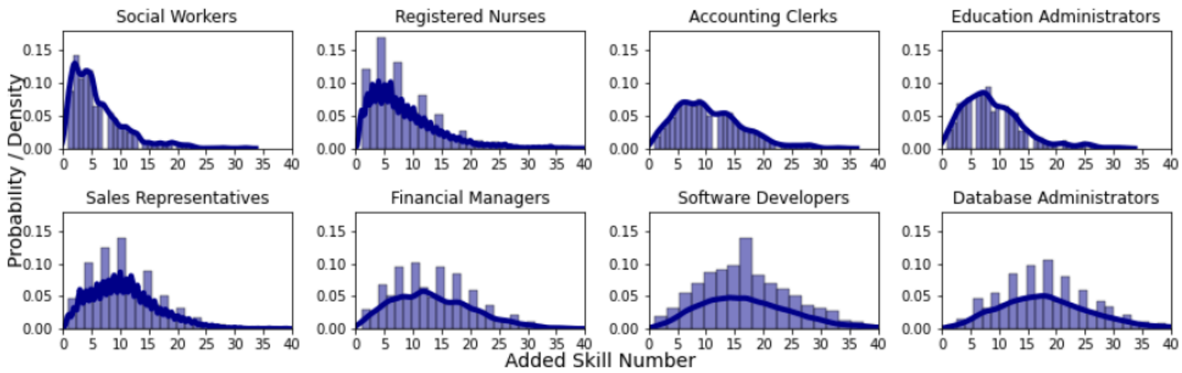

Supplementary Figure 9. Distribution of number of added skills by occupation. Each panel is constructed based on all pairs of within-occupation 2010 job posts, *x* and *y*, where *y* encompasses all skills required by *x* plus additional ones, and meanwhile has a higher education degree requirement than *x*. Each panel presents the distribution of the number of added skills in *y* compared to *x* from all pairs of *x* and *y* under the title occupation: Social Workers (n=44261pairs), Registered Nurses (n=1918593 pairs), Accounting Clerks (n=24723 pairs), Education Administrators (n=7695 pairs), Sales Representatives (n=2035649 pairs), Financial Managers (n=31751 pairs), Software Developers (n=131116 pairs), Database Administrators (n=11320 pairs).

4.10 Figure S10. Education Cost of Marginal Skill Vector Distance between Jobs

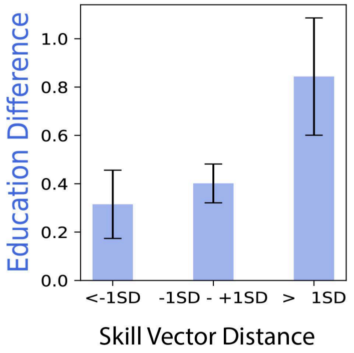

Supplementary Figure 10. Education Cost of Marginal Skill Vector Distance between Jobs. Skill vector distance between jobs corresponds to the amount of re-education cost required for workers to move from one distribution of skills to another. The three bars represent the average required education year difference for pairs of job posts (n=1257 pairs) with different levels of skill vector distances: the first bar consists of pairs with skill vector distances less than 1 standard deviation below the mean, the second bar on pairs with skill vector distances from 1 standard deviation below the mean to one above the mean, and the third bar includes pairs with skill vector distance larger than 1 standard deviation above the mean. Bars represent group mean, and error bars indicate the 95% confidence interval estimated via bootstrapping (50 resamples, each sized at 80% of the original data).

4.11 Figure S11. Unemployment rate and job skill change

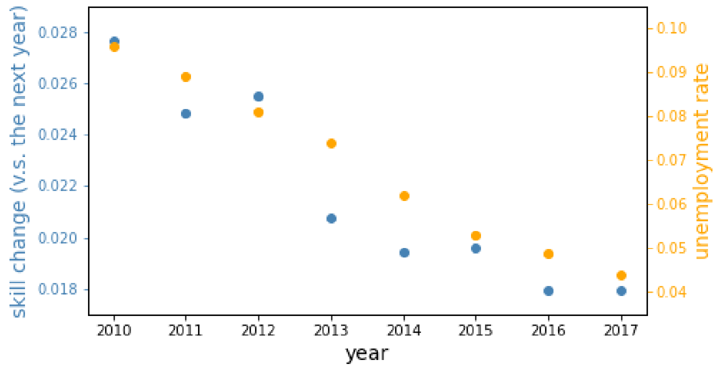

Supplementary Figure 11. Unemployment rate and job skill change. The blue dots represent the year-on-year (e.g., 2010-2011, 2011-2012, etc.) average occupational skill change (n=724 occupations), corresponding to the blue y-axis on the left. The orange dots represent the yearly unemployment rate in the U.S., corresponding to the orange y-axis on the right.

4.12 Figure S12. Mapping U.S. Labor Markets' Upskilling Pressure Through Automation Risk.

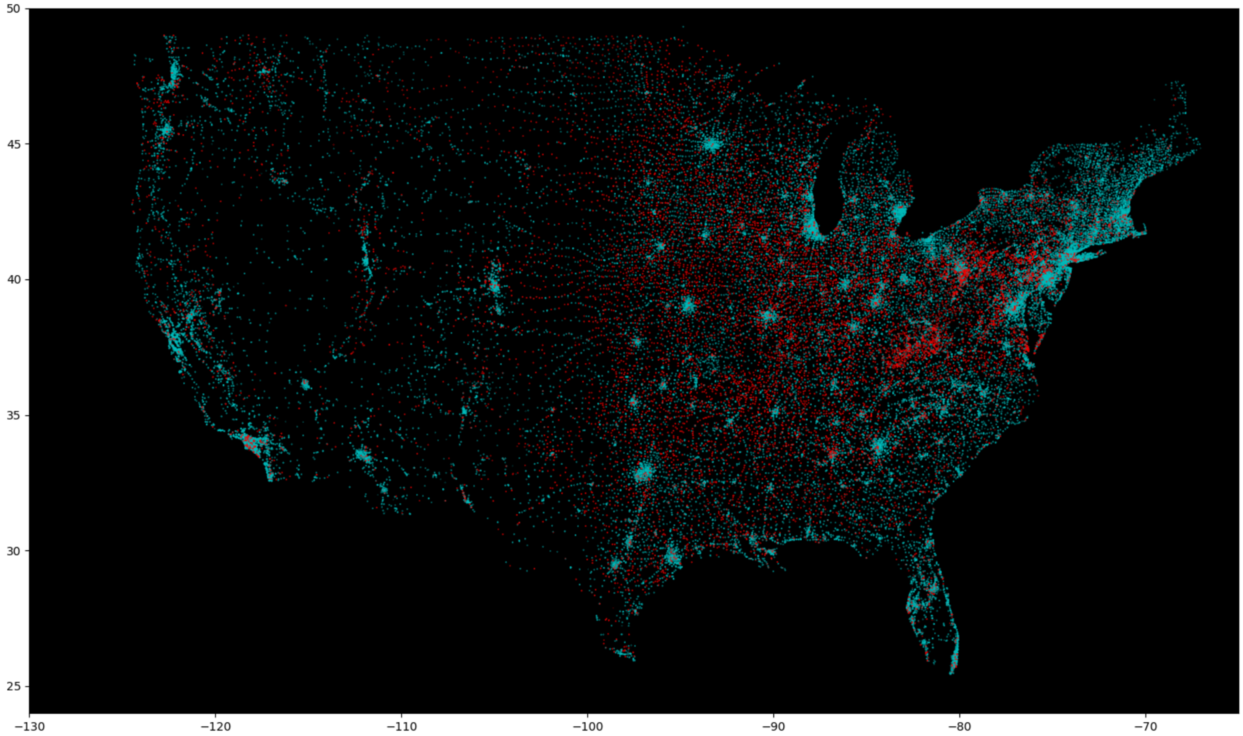

Supplementary Figure 12. Mapping U.S. Labor Markets' Upskilling Pressure Through Automation Risk. This figure integrates two datasets to visualize where future skill-adjustment pressures may concentrate. The Lightcast job-posting dataset (formerly Burning Glass) contains detailed U.S. job advertisements representing 1,060 ONET occupations across 52,979 locations. For this visualization, we use job ads data from 2010 to mid 2019 to calculate local labor demand. The Occupation Automation Risk dataset (OC) provides automation-risk scores for 702 ONET occupations, inferred from a training set of 70 occupations labeled as "computerisable" by artificial-intelligence experts (Frey & Osborne 2017). We map 47,990 U.S. locations with at least three job postings in the selected years. For each location, we construct a local labor-market profile by calculating the occupation share distribution from its observed job postings, then compute its automation-risk score using a market-share-weighted average of OC values. Log average yearly labor-market size and automation risk exhibit a modest but statistically significant negative correlation (Pearson $r = -0.11$, $P < 0.001$), revealing that smaller labor markets face disproportionately higher exposure to computerisation—and thus greater potential upskilling pressure. Locations are color-coded by automation risk—brown for high-risk areas (risk > 0.65; top 25%) and cyan for low-risk areas (≤ 0.65; bottom 75%).

5. Supplementary Tables

5.1 Table S1. Explaining job skill change variation with skill complexity, measuring change with the most significant skill transition

	Dependent variable: Most Significant Skill Transition								
	Max	Max	Max	Rank 5%	Rank 5%	Rank 5%	Rank 10%	Rank 10%	Rank 10%
Skill Complexity	-0.010			-0.053			-0.062		
	(0.002) [p<0.001]			(0.002) [p<0.001]			(0.002) [p<0.001]		
Log Annual Pay			-0.021			-0.053			-0.069
			(0.006) [p<0.001]			(0.007) [p<0.001]			(0.007) [p<0.001]
Education		-0.004			-0.009			-0.010	
		(0.001) [p<0.001]			(0.001) [p<0.001]			(0.001) [p<0.001]	
Obs.	721	721	721	721	721	721	721	721	721
R^2	0.025	0.025	0.019	0.486	0.072	0.081	0.536	0.086	0.113
Adjusted R^2	0.024	0.024	0.018	0.485	0.071	0.080	0.535	0.085	0.112
Residual Std. Error	0.057 (df=719)	0.057 (df=719)	0.057 (df=719)	0.050 (df=719)	0.068 (df=719)	0.067 (df=719)	0.053 (df=719)	0.074 (df=719)	0.073 (df=719)
F Statistic	18.775 (df=1; 719) [p<0.001]	18.379 (df=1; 719) [p<0.001]	14.079 (df=1; 719) [p<0.001]	679.363 (df=1; 719) [p<0.001]	55.877 (df=1; 719) [p<0.001]	63.626 (df=1; 719) [p<0.001]	829.453 (df=1; 719) [p<0.001]	67.906 (df=1; 719) [p<0.001]	91.385 (df=1; 719) [p<0.001]

Notes: Significance of individual coefficients was determined using a two-sided t-test, and overall model significance was assessed with an F-test. P-values are reported without adjustment for multiple comparisons.

5.2 Table S2. Example of job posts pairs used for predicting re-education cost

	Job Ad *i*	Job Ad j	Job Ad *i*	Job Ad *j*
BGTJobId	311043099	311477284	311765112	311777227
Title	Administrative Assistant	Administrative Assistant III	Registered Nurse Signature Suites Vip	Registered Nurse Interventional Radiology N
Education Years	14	16	14	16
Common Skills	Communication Skills<br>Microsoft Excel<br>Microsoft Powerpoint<br>Administrative Support<br>Microsoft Word	Communication Skills<br>Microsoft Excel<br>Microsoft Powerpoint<br>Administrative Support<br>Microsoft Word	Budgeting<br>Patient Care<br>Process Improvement<br>Research<br>Teamwork/Collaboration<br>Customer Contact<br>Life Support<br>Planning	Budgeting<br>Patient Care<br>Process Improvement<br>Research<br>Teamwork/Collaboration<br>Customer Contact<br>Life Support<br>Planning
Additional skill		Customer Information Control System (CICS)<br>Direct Mail<br>Newsletters<br>Portal Tools<br>Siebel CRM		Advanced Cardiac Life Support (ACLS)<br>Catheterization<br>Catheterization Laboratory (CATH LAB)<br>Critical Care<br>Interventional Radiology (IR)
Skill Distance	0.17		0.15	

5.3 Table S3. Predicting education year difference with skill distance

	Dependent variable: Education Year Difference		
	Job Post Unit	Occupation Aggregated Unit	Occupation Aggregated Unit
Skill Distance	0.741	1.839	
	(0.007)<br>[p<0.001]	(0.352)<br>[p<0.001]	
Skill Distance Z-score			0.172
			(0.033)<br>[p<0.001]
Observations	6585524	1257	1257
R^2	0.002	0.021	0.021
Adjusted R^2	0.002	0.020	0.020
Residual Std. Error	2.038 (df=6585522)	1.167 (df=1255)	1.167 (df=1255)
F Statistic	11524.516 (df=1; 6585522)<br>[p<0.001]	27.230 (df=1; 1255)<br>[p<0.001]	27.230 (df=1; 1255)<br>[p<0.001]

Notes: Significance of individual coefficients was determined using a two-sided t-test, and overall model significance was assessed with an F-test. P-values are reported without adjustment for multiple comparisons, shown below standard errors.

5.4 Table S4. Explaining occupation skill change variation with skill complexity

	Dependent variable: Occupation skill change					
	Model 1	Model 2	Model 3	Model 4	Model 5	Model 6
Skill Complexity	-0.012			-0.012		
	(0.001)<br>[p<0.001]			(0.001)<br>[p<0.001]		
Log Annual Pay		-0.007			-0.005	
		(0.001)<br>[p<0.001]			(0.001)<br>[p<0.001]	
Education			-0.001			-0.002
			(0.000)<br>[p<0.001]			(0.000)<br>[p<0.001]
Log Emp. Concentr.				0.001	0.013	0.015
				(0.001)<br>[p=0.422]	(0.001)<br>[p<0.001]	(0.001)<br>[p<0.001]
Job Similarity Increase				-0.030	-0.027	-0.022
				(0.012)<br>[p=0.013]	(0.013)<br>[p=0.042]	(0.013)<br>[p=0.091]
Job Similarity				-0.027	-0.022	-0.014
				(0.007)<br>[p<0.001]	(0.007)<br>[p=0.002]	(0.007)<br>[p=0.066]
Observations	668	668	668	668	668	668
R^2	0.300	0.037	0.033	0.319	0.178	0.199
Adjusted R^2	0.299	0.035	0.032	0.315	0.173	0.194
Residual Std. Error	0.046 (df=666)	0.058 (df=666)	0.058 (df=666)	0.045 (df=663)	0.052 (df=663)	0.052 (df=663)
F Statistic	284.882 (df=1; 666) [p<0.001]	25.304 (df=1; 666) [p<0.001]	22.842 (df=1; 666) [p<0.001]	77.807 (df=4; 663) [p<0.001]	35.959 (df=4; 663) [p<0.001]	41.258 (df=4; 663) [p<0.001]

Notes: Significance of individual coefficients was determined using a two-sided t-test, and overall model significance was assessed with an F-test. P-values are reported without adjustment for multiple comparisons. Skill complexity refers to the log occupational core skill number averaged over 2010 and 2018. Log annual pay refers to the log value of occupation average annual median pay. Education refers to the occupation average education year requirements. Log Emp. Concentr. refers to the natural log of HHI (employer concentration). Job similarity measures how similar job posts are within the same occupation in a given year. Job similarity increase captures the difference between 2018 and 2010 within-occupation job homogeneity level.

5.5 Table S5. Explaining skill change variation with employer and market size, within firm-region-occupation change

	Dependent variable: Firm-region-occupation skill change				
	Model 1	Model 2	Model 3	Model 4	Model 5
Firm Size	-0.004		-0.003		-0.002
	(0.001)<br>[p<0.001]		(0.001)<br>[p<0.001]		(0.001)<br>[p=0.065]
CZ Market Size		-0.008		-0.022	-0.022
		(0.001)<br>[p<0.001]		(0.002)<br>[p<0.001]	(0.002)<br>[p<0.001]
Log Emp. Concentr.			-0.005	-0.021	-0.021
			(0.001)<br>[p<0.001]	(0.002)<br>[p<0.001]	(0.002)<br>[p<0.001]
Job Similarity Increase			0.099	0.102	0.099
			(0.015)<br>[p<0.001]	(0.015)<br>[p<0.001]	(0.015)<br>[p<0.001]
Occ. FE	Yes	Yes	Yes	Yes	Yes
Observations	4497	4497	4497	4497	4497
R^2	0.113	0.116	0.124	0.150	0.151
Adjusted R^2	0.088	0.091	0.099	0.126	0.126
Residual Std. Error	0.084 (df=4374)	0.084 (df=4374)	0.084 (df=4372)	0.083 (df=4372)	0.083 (df=4371)
F Statistic	4.569 (df=122; 4374) [p<0.001]	4.700 (df=122; 4374) [p<0.001]	4.987 (df=124; 4372) [p<0.001]	6.227 (df=124; 4372) [p<0.001]	6.208 (df=125; 4371) [p<0.001]

Notes: Significance of individual coefficients was determined using a two-sided t-test, and overall model significance was assessed with an F-test. P-values are reported without adjustment for multiple comparisons. CZ refers to the commuting zone. Firm and CZ market size are their corresponding log number of job posts averaged over 2010 and 2018. Log Emp. Concentr. refers to the natural log of HHI (employer concentration). Job similarity increase measures the difference between 2018 and 2010 within-firm-cz-occupation job homogeneity level.

5.6 Table S6. Explaining skill change variation with employer and market size, firm-region overall change

	Dependent variable: Firm-region skill change				
	Model 1	Model 2	Model 3	Model 4	Model 5
Firm Size	-0.001		-0.001		-0.001
	(0.000) [p=0.009]		(0.000) [p=0.025]		(0.000) [p=0.030]
CZ Market Size		-0.003		-0.019	-0.019
		(0.001) [p<0.001]		(0.004) [p<0.001]	(0.004) [p<0.001]
Log Emp. Concentr.			0.012	-0.066	-0.066
			(0.003) [p<0.001]	(0.015) [p<0.001]	(0.015) [p<0.001]
Job Similarity Increase			-0.005	-0.004	-0.001
			(0.018) [p=0.785]	(0.018) [p=0.837]	(0.018) [p=0.945]
Observations	13141	13141	13141	13141	13141
R^2	0.001	0.002	0.002	0.004	0.004
Adjusted R^2	0.000	0.002	0.002	0.004	0.004
Residual Std. Error	0.109 (df=13139)	0.109 (df=13139)	0.109 (df=13137)	0.109 (df=13137)	0.108 (df=13136)
F Statistic	6.731 (df=1; 13139) [p=0.009]	30.511 (df=1; 13139) [p<0.001]	8.941 (df=3; 13137) [p<0.001]	16.793 (df=3; 13137) [p<0.001]	13.776 (df=4; 13136) [p<0.001]

Notes: Significance of individual coefficients was determined using a two-sided t-test, and overall model significance was assessed with an F-test. P-values are reported without adjustment for multiple comparisons. CZ refers to the commuting zone. Firm and CZ market size are their corresponding log number of job posts averaged over 2010 and 2018. Log Emp. Concentr. refers to the natural log of HHI (employer concentration). Job similarity increase measures the difference between 2018 and 2010 firm-cz average of within-firm-cz-occupation job homogeneity level.

5.7 Table S7. Explaining occupation skill change variation with skill complexity, different job content scope and skill weights

	Dependent variable: Occupation skill change		
	All Skills	Top 50% Skills	Top 25% Skills
Skill Complexity	-0.006	-0.007	-0.007
	(0.001)<br>[p<0.001]	(0.001)<br>[p<0.001]	(0.001)<br>[p<0.001]
Log Emp. Concentr.	0.003	0.003	0.003
	(0.002)<br>[p=0.046]	(0.002)<br>[p=0.042]	(0.002)<br>[p=0.034]
Job Similarity Increase	-0.050	-0.051	-0.053
	(0.012)<br>[p<0.001]	(0.012)<br>[p<0.001]	(0.013)<br>[p<0.001]
Job Similarity	-0.029	-0.030	-0.031
	(0.007)<br>[p<0.001]	(0.007)<br>[p<0.001]	(0.007)<br>[p<0.001]
Observations	668	668	668
R^2	0.171	0.175	0.183
Adjusted R^2	0.166	0.170	0.178
Residual Std. Error	0.031 (df=663)	0.032 (df=663)	0.036 (df=663)
F Statistic	34.129 (df=4; 663)<br>[p<0.001]	35.044 (df=4; 663)<br>[p<0.001]	37.004 (df=4; 663)<br>[p<0.001]

Notes: Significance of individual coefficients was determined using a two-sided t-test, and overall model significance was assessed with an F-test. P-values are reported without adjustment for multiple comparisons. Skill complexity refers to the log occupational core skill number averaged over 2010 and 2018. Log Emp. Concentr. refers to the natural log of HHI (employer concentration). Job similarity measures how similar job posts are within the same firm, market, occupation in a given year.

5.8 Table S8. Explaining skill change variation with employer and market size (within firm-region-occupation change), different job content scope and skill weights

	Dependent variable: Firm-region-occupation skill change		
	All Skills	Top 50% Skills	Top 25% Skills
Firm Size	-0.005	-0.006	-0.006
	(0.000)<br>[p<0.001]	(0.000)<br>[p<0.001]	(0.000)<br>[p<0.001]
CZ Market Size	-0.009	-0.011	-0.013
	(0.000)<br>[p<0.001]	(0.000)<br>[p<0.001]	(0.000)<br>[p<0.001]
Log Emp. Concentr.	-0.007	-0.010	-0.013
	(0.000)<br>[p<0.001]	(0.000)<br>[p<0.001]	(0.000)<br>[p<0.001]
Job Similarity Increase	0.000	0.003	0.009
	(0.001)<br>[p=0.961]	(0.001)<br>[p=0.032]	(0.001)<br>[p<0.001]
Occ. FE	Yes	Yes	Yes
Observations	283151	283151	283151
R^2	0.138	0.146	0.155
Adjusted R^2	0.136	0.144	0.153
Residual Std. Error	0.104 (df=282484)	0.108 (df=282484)	0.116 (df=282484)
F Statistic	68.046 (df=666; 282484)<br>[p<0.001]	72.578 (df=666; 282484)<br>[p<0.001]	77.927 (df=666; 282484)<br>[p<0.001]

Notes: Significance of individual coefficients was determined using a two-sided t-test, and overall model significance was assessed with an F-test. P-values are reported without adjustment for multiple comparisons. CZ refers to the commuting zone. Firm and CZ market size are their corresponding log number of job posts averaged over 2010 and 2018. Log Emp. Concentr. refers to the natural log of HHI (employer concentration). Job similarity measures how similar job posts are within the same occupation in a given year. Job similarity increase captures the difference between 2018 and 2010 within-occupation job homogeneity level.

5.9 Table S9. Explaining skill change variation with employer and market size (firm-region overall change), different job content scope and skill weights

	Dependent variable: Firm-region skill change		
	All Skills	Top 50% Skills	Top 25% Skills
Firm Size	-0.009	-0.009	-0.010
	(0.000)<br>[p<0.001]	(0.000)<br>[p<0.001]	(0.000)<br>[p<0.001]
CZ Market Size	-0.023	-0.027	-0.030
	(0.001)<br>[p<0.001]	(0.001)<br>[p<0.001]	(0.001)<br>[p<0.001]
Log Emp. Concentr.	-0.058	-0.066	-0.068
	(0.004)<br>[p<0.001]	(0.004)<br>[p<0.001]	(0.005)<br>[p<0.001]
Job Similarity Increase	-0.146	-0.141	-0.132
	(0.004)<br>[p<0.001]	(0.004)<br>[p<0.001]	(0.005)<br>[p<0.001]
Observations	143934	143934	143934
R^2	0.073	0.073	0.077
Adjusted R^2	0.073	0.073	0.077
Residual Std. Error	0.134 (df=143929)	0.137 (df=143929)	0.146 (df=143929)
F Statistic	2838.982 (df=4; 143929)<br>[p<0.001]	2838.661 (df=4; 143929)<br>[p<0.001]	2993.067 (df=4; 143929)<br>[p<0.001]

Notes: Significance of individual coefficients was determined using a two-sided t-test, and overall model significance was assessed with an F-test. P-values are reported without adjustment for multiple comparisons. CZ refers to the commuting zone. Firm and CZ market size are their corresponding log number of job posts averaged over 2010 and 2018. Log Emp. Concentr. refers to the natural log of HHI (employer concentration). Job similarity Increase measures the difference between 2018 and 2010 firm-cz average of within-firm-cz-occupation job homogeneity level.

References

1. Lancaster, V., Mahoney-Nair, D. & Ratcliff, N. J. Review of burning glass job-ad data. https://biocomplexity.virginia.edu/sites/default/files/projects/Technical%20Report%20Review%20of%20BGT%20Job-ad%20Data.pdf (2019).
2. Deming, D. J. & Noray, K. Earnings Dynamics, Changing Job Skills, and STEM Careers*. *The Quarterly Journal of Economics* vol. 135 1965–2005 (2020).
3. Anderson, K. A. Skill networks and measures of complex human capital. *Proc. Natl. Acad. Sci. U. S. A.* **114**, 12720–12724 (2017).
4. van Dam, A., Gomez-Lievano, A., Neffke, F. & Frenken, K. An information-theoretic approach to the analysis of location and co-location patterns. *arXiv [stat.AP]* (2020).
5. Newman, M. E. J. & Girvan, M. Finding and evaluating community structure in networks. *Physical Review E* vol. 69 (2004).
6. Kim, S., Ahn, Y.-Y. & Park, J. Labor space: A unifying representation of the labor market via large language models. in *Proceedings of the ACM Web Conference 2024* vol. 358 2441–2451 (ACM, New York, NY, USA, 2024).
7. Neffke, F., Nedelkoska, L. & Wiederhold, S. Skill mismatch and the costs of job displacement. *Res. Policy* **53**, 104933 (2024).
8. Poletaev, M. & Robinson, C. Human capital specificity: Evidence from the dictionary of occupational titles and displaced worker surveys, 1984–2000. *J. Labor Econ.* **26**, 387–420 (2008).
9. Arora, S., Li, Y., Liang, Y., Ma, T. & Risteski, A. Linear algebraic structure of word senses, with applications to polysemy. *Transactions of the Association for Computational Linguistics* **6**, 483–495 (2018).
10. Arseniev-Koehler, A., Cochran, S. D., Mays, V. M., Chang, K.-W. & Foster, J. G. Integrating topic modeling and word embedding to characterize violent deaths. *Proc. Natl. Acad. Sci. U. S. A.* **119**, e2108801119 (2022).
11. Devereux, P. J. Occupational upgrading and the business cycle. *Labour* **16**, 423–452 (2002).
12. Modestino, A. S., Shoag, D. & Ballance, J. Downskilling: changes in employer skill requirements over the business cycle. *Labour Econ.* **41**, 333–347 (2016).
13. Modestino, A. S., Shoag, D. & Ballance, J. Upskilling: Do employers demand greater skill when skilled workers are plentiful? *SSRN Electron. J.* (2015) doi:10.2139/ssrn.2788601.

14. Schubert, G., Stansbury, A. & Taska, B. Employer Concentration and Outside Options. (2022) doi:10.2139/ssrn.3599454.

15. Azar, J., Marinescu, I. & Steinbaum, M. Labor Market Concentration. *J. Hum. Resour.* **57**, S167–S199 (2022).

16. Hershbein, B., Macaluso, C. & Yeh, C. Concentration in U.S. local labor markets: evidence from vacancy and employment data. https://economicdynamics.org/meetpapers/2019/paper_1336.pdf (2019).
