# Supplementary material for: Lower-skilled occupations face greater upskilling pressure in U.S. job ads
Source: Nat Commun. 2025 Dec 31;17:1237. doi: 10.1038/s41467-025-67992-y (PMC12864811; doi:10.1038/s41467-025-67992-y)
Supplement: Supplementary file 2 — Reporting Summary [file 41467_2025_67992_MOESM2_ESM.pdf]

nature portfolio

Corresponding author(s): *Double-anonymous peer review submissions: write DAPR and your manuscript number here instead of author names.*

Last updated by author(s): Nov 28, 2025

Reporting Summary

Nature Portfolio wishes to improve the reproducibility of the work that we publish. This form provides structure for consistency and transparency in reporting. For further information on Nature Portfolio policies, see our Editorial Policies and the Editorial Policy Checklist.

Statistics

For all statistical analyses, confirm that the following items are present in the figure legend, table legend, main text, or Methods section.

n/a	Confirmed	
☐	☒	The exact sample size (*n*) for each experimental group/condition, given as a discrete number and unit of measurement
☐	☒	A statement on whether measurements were taken from distinct samples or whether the same sample was measured repeatedly
☐	☒	The statistical test(s) used AND whether they are one- or two-sided<br>*Only common tests should be described solely by name; describe more complex techniques in the Methods section.*
☐	☒	A description of all covariates tested
☐	☒	A description of any assumptions or corrections, such as tests of normality and adjustment for multiple comparisons
☐	☒	A full description of the statistical parameters including central tendency (e.g. means) or other basic estimates (e.g. regression coefficient) AND variation (e.g. standard deviation) or associated estimates of uncertainty (e.g. confidence intervals)
☐	☒	For null hypothesis testing, the test statistic (e.g. *F*, *t*, *r*) with confidence intervals, effect sizes, degrees of freedom and *P* value noted<br>*Give P values as exact values whenever suitable.*
☒	☐	For Bayesian analysis, information on the choice of priors and Markov chain Monte Carlo settings
☐	☒	For hierarchical and complex designs, identification of the appropriate level for tests and full reporting of outcomes
☐	☒	Estimates of effect sizes (e.g. Cohen's *d*, Pearson's *r*), indicating how they were calculated

Our web collection on statistics for biologists contains articles on many of the points above.

Software and code

Policy information about availability of computer code

Data collection	Proprietary data provided by Lightcast (formerly known as Burning Glass Technologies).
Data analysis	Data analysis was conducted with python, all codes available at https://github.com/di-Tong/SkillPaper/tree/master/Codes

For manuscripts utilizing custom algorithms or software that are central to the research but not yet described in published literature, software must be made available to editors and reviewers. We strongly encourage code deposition in a community repository (e.g. GitHub). See the Nature Portfolio guidelines for submitting code & software for further information.

Data

Policy information about availability of data

All manuscripts must include a data availability statement. This statement should provide the following information, where applicable:

- Accession codes, unique identifiers, or web links for publicly available datasets
- A description of any restrictions on data availability
- For clinical datasets or third party data, please ensure that the statement adheres to our policy

The raw job posting data used in this study are available under restricted access from LightCast; access can be obtained through a licensing agreement with LightCast, with details at https://lightcast.io/. The processed data generated in this study, including aggregated occupation-year level skill demands and skill embeddings derived from job postings, are available at https://github.com/di-Tong/SkillPaper/tree/master/IntermediateData (archived on Zenodo under https://doi.org/10.5281/zenodo.17444902). A comprehensive variable dictionary for LightCast data is provided in the Supplementary Information and at https://github.com/di-Tong/SkillPaper/tree/master/Codes.

The publicly available datasets used in this study are available as follows: the 2010 Penn State University Labor-Sheds for Regional Analysis data at https://sites.psu.edu/psucz/data/, the 2018 CPS data at https://cps.ipums.org/cps/, O*NET job zone data at https://www.onetonline.org/, 2010 and 2018 BLS Occupational Employment Statistics (OES) data at https://www.bls.gov/oes/tables.htm, and Labor Force Statistics data at https://www.bls.gov/cps/cps_aa2018.htm.
For further inquiries regarding data access, researchers may contact LightCast directly or reach out to the corresponding author.

Research involving human participants, their data, or biological material

Policy information about studies with human participants or human data. See also policy information about sex, gender (identity/presentation), and sexual orientation and race, ethnicity and racism.

Reporting on sex and gender	Our research does not involve human participants or collect individual level data. We study occupational level skill change with job advertisement data that do not have information on sex or gender. To understand the implication of skill change on workers of different social groups, we associate occupational skill change with occupational demographic composition, leveraging occupational employment by sex statistics from the U.S. Bureau of Labor Statistics based on the 2018 Current Population Survey, a survey of households conducted by the Bureau of Census. The Current Population Survey is designed to identify biological sex.
Reporting on race, ethnicity, or other socially relevant groupings	Our research does not involve human participants or collect individual level data. We study occupational level skill change with job advertisement data that do not have information on race or ethnicity. To understand the implication of skill change on workers of different social groups, we associate occupational skill change with occupational demographic composition, leveraging occupational employment by race statistics from the U.S. Bureau of Labor Statistics based on the 2018 Current Population Survey, a survey of households conducted by the Bureau of Census. The Current Population Survey asks respondents to indicate their races or races they consider themselves to be.
Population characteristics	Not applicable
Recruitment	Not applicable
Ethics oversight	Not applicable

Note that full information on the approval of the study protocol must also be provided in the manuscript.

Field-specific reporting

Please select the one below that is the best fit for your research. If you are not sure, read the appropriate sections before making your selection.

☐ Life sciences ☒ Behavioural & social sciences ☐ Ecological, evolutionary & environmental sciences

For a reference copy of the document with all sections, see nature.com/documents/nr-reporting-summary-flat.pdf

Behavioural & social sciences study design

All studies must disclose on these points even when the disclosure is negative.

Study description	Quantitative observational study with large-scale text data and natural language processing approach
Research sample	We use a dataset of more than 167 million job ads from 2010 to 2018 provided by Lightcast (formerly known as Burning Glass Technologies). This is the best available data for dynamic skill requirements across jobs, employers, and regions. This dataset has been used and validated with other labor market data source by a number of papers published on top economics and management journals.
Sampling strategy	The sample covers a near universe of online job advertisements in U.S. labor market from 2010-2018.
Data collection	Lightcast collects information from more than 40,000 job boards and company websites daily.
Timing	2010-2018
Data exclusions	2007-2009 data has been excluded because data during that period is very sparse and incomplete.
Non-participation	Not applicable
Randomization	Not applicable

Reporting for specific materials, systems and methods

We require information from authors about some types of materials, experimental systems and methods used in many studies. Here, indicate whether each material, system or method listed is relevant to your study. If you are not sure if a list item applies to your research, read the appropriate section before selecting a response.

Materials & experimental systems

n/a	Involved in the study	
☒	☐	Antibodies
☒	☐	Eukaryotic cell lines
☒	☐	Palaeontology and archaeology
☒	☐	Animals and other organisms
☒	☐	Clinical data
☒	☐	Dual use research of concern
☒	☐	Plants

Methods

n/a	Involved in the study	
☒	☐	ChIP-seq
☒	☐	Flow cytometry
☒	☐	MRI-based neuroimaging

Plants

Seed stocks	Not applicable
Novel plant genotypes	Not applicable
Authentication	Not applicable
